# Communication in the infodemic: building communication competencies for effective public health leadership

**DOI:** 10.1093/eurpub/ckae190

**Published:** 2025-03-25

**Authors:** Seema Yasmin

**Affiliations:** Department of Medicine, Stanford University, Stanford, CA, United States

On July 9, 1999, the United States Public Health Service and the American Academy of Pediatrics asked US vaccine makers to remove thimerosal, a mercury-containing preservative, from vaccines [1]. That same day, the AAP issued a statement, which included the following language: “The current levels of thimerosal will not hurt children, but reducing those levels will make safe vaccines even safer. While our current immunization strategies are safe, we have an opportunity to increase the margin of safety.”

The consequences of this messaging were devastating. The decision to remove thimerosal—an antibacterial preservative used in medicines since the 1930s, and long-stated to be safe by agencies including the Centers for Disease Control and Prevention and the Food and Drug Administration—signaled to concerned parents that thimerosal must be unsafe: why else would the nation’s leading public health agency demand its removal? Anti-vaccine groups were founded on the core belief that this decision to remove thimerosal from vaccines was proof that the medical establishment had long-known thimerosal caused harm to children [2].

Doctors were confused by the messaging [3]. They were asked to delay the birth dose of the hepatitis B vaccine in newborns not considered at risk of infection while vaccine makers were in the process of removing thimerosal. But 1 in 10 hospitals suspended use of the vaccine altogether, and at least one infant, a baby girl in Michigan born to a mother who tested positive for hepatitis B, died from liver failure caused by fulminant infection [4].

When we talk about health communication with negative consequences, we often focus on false information spread by bad actors who work outside of public health agencies. Indeed, scientific disinformation has become an area of considerable study since the emergence of SARS-CoV-2. But what about the many instances of communication, designed and disseminated not by bad actors but by well-meaning public health agencies, which have caused confusion and harm?

During a lecture at the World Health Organization’s inaugural European Public Health Leadership Course in 2022, I began by defining information disorder—an umbrella term that includes misinformation and disinformation—and reviewing the thimerosal case study. I asked the public health leaders present to subcategorize this example of messaging-gone-wrong: Was it misinformation—false information unknowingly spread by well-meaning people? Or was it disinformation—information known to be false and spread with bad intent?

It was neither. The messaging was based on accurate information. Vaccine makers were in fact being asked to remove thimerosal, and healthcare workers were being asked to institute a protocol change during the interim period. Besides the fact that the decision was made on shaky scientific ground (there was little evidence at the time that thimerosal caused harm and numerous studies have since demonstrated its safety), the messaging was factual. This instance of public health messaging, and others like it, fall into the yet-to-be-named category of well-meaning communications from benevolent but misguided messengers.

Poorly crafted, unresearched, and untested messaging from public health agencies has the potential to deepen distrust in science and public health, to negatively influence the public’s health behaviors, and result in misunderstanding and injury. So why do we continue to relegate communication to an afterthought; something to be considered only after a vaccine has been developed or a campaign is ready to launch? (Consider that Operation Warp Speed, the U.S. government’s public–private partnership to accelerate the development and distribution of COVID-19 vaccines, diagnostics, and treatments, invested considerable resources in the “hard science” of vaccine-making and testing, but far fewer resources on the “soft science” of developing and testing messaging—despite the success of the endeavor relying upon the public’s agreement to receive vaccines.)

Communication is one of the most ubiquitous public health interventions, but it is not respected as such. Communication is inadequately taught during undergraduate and post-graduate science, public health, and medical training. Despite its ubiquity and potential—indeed, goal—to land considerable impact, communication remains an undervalued competency, misperceived as a skill that requires little or no training to master.

We practice evidence-based medicine but fail to practice evidence-based communications. This, despite a considerable body of scholarship, which, over decades, has dissected, analyzed, and identified considerable limitations in the typical models of public health communication. We turn to outdated, paternalistic, monodirectional modes of communication, such as the knowledge deficit model, which burden the messenger—often a public health leader—with the impossible task of embodying omniscience and infallibility. In other words, we set ourselves up for failure.

While a paradigm shift in undergraduate and postgraduate training will take years to institute, public health agencies can learn from the WHO’s Public Health Leadership Course, which sought to remind public health leaders that communication is a science; that skills of trust-building, deep listening, and effective messaging can be taught; and that dedicated time to study the latest evidence-based methods for countering falsehoods are essential components of leadership.

Leadership trainings for public health officials must incorporate lessons from community listening exercises and sentiment analysis of scientific and public health information and misinformation circulating at the community level. Studies that explore the scalability and feasibility of these programs should be discussed so that public health leaders can translate findings to their regions and implement new models of bidirectional communications.

Trust is at the heart of effective communications. Building trust requires radical transparency, another area that requires a paradigm shift in the way public health leaders communicate with publics. Across agencies and institutions and throughout the world, my recommendation that public health leaders model radical transparency and even admit to error is met with horror. But the archetype of public health leader as omniscient and infallible is a harmful one, both to the publics and those in leadership positions. New ways of communicating can be forged; these skills can be taught. But doing so requires the intentional design of leadership programs that incorporate these lessons alongside opportunities to role play and test them out.

Public health emergencies of the future will always co-exist with infodemics—overwhelming amounts of information, some factual, some false, which exacerbate distrust in science and fuel the kind of fear and panic that can upend even well-designed public health efforts. Public health leaders must be equipped with the skills to not only conduct surveillance on disease but also to conduct surveillance on disinformation about disease. They must know how to work with previously identified networks of trusted messengers to disseminate targeted communications to segmented audiences. They must work to iterate and split-test messaging long before a vaccine, diagnostic, or therapeutic is launched. Failure to employ these methods will only amplify the efforts of the small but powerful anti-public health contingents whose well-researched and sophisticated storytelling can quickly unravel the efforts of years of scientific research and public health planning.

The historic outbreak of measles among Somali-American children in Minnesota in 2017—the largest measles outbreak in Minnesota in three decades—was the result of a year-long and carefully tailored messaging effort by anti-vaccine groups who decimated trust in the MMR vaccine among Somali-American parents [5]. Such crises constitute a failure of public health leadership—a failure based on the dangerous misunderstanding that communication is not a powerful and teachable skill worthy of incorporating into leadership training.

## References

[1] Thimerosal in vaccines: a joint statement of the American Academy of Pediatrics and the Public Health Service. MMWR Morb Mortal Wkly Rep 1999;48:563–5. 10418806

[2] Offit PA. Thimerosal and vaccines—a cautionary tale. N Engl J Med 2007;357:1278–9. 10.1056/NEJMp078187 17898096

[3] Recommendations regarding the use of vaccines that contain thimerosal as a preservative. MMWR Morb Mortal Wkly Rep 1999;48:996–8. https://www.cdc.gov/mmwr/preview/mmwrhtml/mm4843a4.htm 10577494

[4] Connecticut Department of Public Health Immunization Action Plan, Fall 2000. https://portal.ct.gov/-/media/Departments-and-Agencies/DPH/dph/infectious_diseases/immunization/IAP_On_Time/IAPVOL41pdf.pdf (14 August 2023, date last accessed)

[5] Hall V , Banerjee E, Kenyon C et al Measles outbreak—Minnesota April-May 2017. MMWR Morb Mortal Wkly Rep 2017;66:713–7. 10.15585/mmwr.mm6627a1 28704350 PMC5687591

